# Beyond the Impact Factor: Reflecting on Twenty Years of Leading Efforts in Research, Innovation in Publishing, and Investment in People

**DOI:** 10.2196/16390

**Published:** 2019-10-31

**Authors:** John Torous

**Affiliations:** 1 Division of Digital Psychiatry, Department of Psychiatry Beth Israel Deaconess Medical Center Boston, MA United States

**Keywords:** JMIR, publishing, eHealth, digital health, digital medicine, knowledge dissemination

## Abstract

This viewpoint celebrates the accomplishments of the Journal of Medical Internet Research (JMIR) on its twentieth anniversary and reviews accomplishments around research publications, journal innovation, and supporting people.

“Form follows function” is an aphorism that has transcended architecture, computer science, and now even digital health. Reflecting on the twentieth anniversary of the Journal of Medical Internet Research (JMIR), “form follows function” also well explains the success and impact of the journal in promoting high quality research, supporting innovation in publication, and supporting a generation of young investigators.

In the 20 years JMIR has represented the field of digital health, the nature of digital health research has evolved and transformed. Today’s papers often focus on machine learning, virtual reality, smartphone apps, and other topics that were nonexistent or nascent in health care research in 1999 when the journal began, or in 2001 when the journal outlined a clear definition for electronic health (eHealth) [1]. Yet today, the journal continues to function as a hub for digital health research. The prescient nature of JMIR in publishing not only high quality but also innovative articles is exemplified by a sample of papers from 20 years prior, with topics including evaluation of consumer health tools [2], neural nets for medical decision making [3], and online prescribing [4], all of which were published in 1999. With its reputation for high quality publications, the journal has offered clinicians, patients, researchers, entrepreneurs, and policy makers a window into best practices and current evidence for digital health. While it is impossible to know what the critical topics and forms of research in digital health will be in the future, it is possible to state that JMIR will cover those with the same high standards and functions it has stood by for its first 20 years.

However, JMIR is more than high quality research. The interdisciplinary nature, rapid paradigm shifts, and global impact of digital health requires a journal to adopt a unique form to meet its function in this space. For 20 years, JMIR has been rethinking how a journal can support the digital mental health community with leading efforts in open access publication, preprints, an easy to access Web and mobile layout, social media outreach and dissemination, crowdfunding, and offering peer reviewers publication credits, among others. The journal’s adaptable online format can support research from not only the health fields but also engineering, computer sciences, design, implementation, policy, and other unique disciplines. Its unique functioning has created a form that offers the ability to publish unique special issues [5] on topics that otherwise would likely not find a voice. The format of JMIR has adapted with changes in the way people access, share, and read research papers. New innovations such as the new JMIRx journal series ("superjournals" or overlay journals for Preprint servers, announced in this 20th Anniversary Theme Issue), highlight ongoing efforts to expand the form of the journal towards an open science platform.

Even beyond high quality research and innovative publishing models, JMIR’s legacy of 20 years includes supporting people. The journal has forged a reputation for offering patients a voice in the medical world [6,7]. It also serves as a forum to engage all members of the digital health ecosystem to share their research, perspectives, and ideas, whether as a world-leading research team [8] or simply as a person with a thoughtful and important perspective to share. On a personal note, the journal has supported my own career in digital health, offering a venue to share my early research in 2014 [9] and later to serve on the editorial board, before then becoming editor-in-chief of JMIR Mental Health. I can also count many other junior investigators for whom JMIR has served as a mentor and adapted its form to help support their interests and exploration of digital health. This commitment towards supporting people of all natures and backgrounds may be the most enduring and impressive aspect of the first 20 years of JMIR.

Reflecting on the twentieth anniversary of JMIR, it is clear that “form follows function” only begins to describe the innovation and impact of the journal. From leading efforts in research, innovation in publication, and investment in people, the field has grown because of JMIR and its support for digital health. While the format of JMIR will continue to adapt to lead the field, its function as a leading force will remain constant and it will continue to act as a beacon of certainty in the always evolving world of digital health.

## Abbreviations

eHealth: electronic health
JMIR: Journal of Medical Internet Research

## References

[1] Eysenbach G (2001). What is e-health?. J Med Internet Res.

[2] Cui L (1999). Rating health web sites using the principles of citation analysis: a bibliometric approach. J Med Internet Res.

[3] Kanagaratnam B, Lavelle S, Comerford R (1999). FTO1/362: Using Neural Nets in Medical Decision Making. J Med Internet Res.

[4] Eysenbach G (1999). Online prescribing of sildanefil (Viagra) on the world wide web. J Med Internet Res.

[5] Calvo RA, Dinakar K, Picard R, Christensen H, Torous J (2018). Toward Impactful Collaborations on Computing and Mental Health. J Med Internet Res.

[6] Torous J, Roux S (2017). Patient-Driven Innovation for Mobile Mental Health Technology: Case Report of Symptom Tracking in Schizophrenia. JMIR Ment Health.

[7] Chiauzzi E, Newell A (2019). Mental Health Apps in Psychiatric Treatment: A Patient Perspective on Real World Technology Usage. JMIR Ment Health.

[8] Bosl W, Mandel J, Jonikas M, Ramoni RB, Kohane IS, Mandl KD (2013). Scalable decision support at the point of care: a substitutable electronic health record app for monitoring medication adherence. Interact J Med Res.

[9] Torous J, Friedman R, Keshavan M (2014). Smartphone ownership and interest in mobile applications to monitor symptoms of mental health conditions. JMIR Mhealth Uhealth.

